# Sex and Body Mass Index Differences in Changes in Skin Temperature After Repeated Sessions of Whole-Body Cryostimulation

**DOI:** 10.3390/jcm13237365

**Published:** 2024-12-03

**Authors:** Paolo Piterà, Raffaella Cancello, Jacopo Maria Fontana, Federica Verme, Romain Bouzigon, Benoit Dugué, Amelia Brunani, Paolo Capodaglio

**Affiliations:** 1 Department of Neurosciences “Rita Levi Montalcini”, University of Turin, 10126 Turin, Italy; p.pitera@auxologico.it; 2 Laboratory of Clinical Neurobiology, IRCCS Istituto Auxologico Italiano, San Giuseppe Hospital, 28824 Verbania, Italy; 3 Obesity Unit, Laboratory of Nutrition and Obesity Research, Department of Endocrine and Metabolic Diseases, IRCCS Istituto Auxologico Italiano, 20149 Milan, Italy; r.cancello@auxologico.it; 4 Research Laboratory in Biomechanics, Rehabilitation and Ergonomics, IRCCS Istituto Auxologico Italiano, San Giuseppe Hospital, 28824 Verbania, Italy; f.verme@auxologico.it (F.V.); brunani@auxologico.it (A.B.); p.capodaglio@auxologico.it (P.C.); 5 Sociètè Inside the Athletes 3.0, Sports Performance Optimization Complex, 25000 Besançon, France; romain.bouzigon@univ-fcomte.fr; 6 Unit of Formation and Research in Sports, Laboratory C3S (EA 4660), Department of Sport and Performance, University of Franche-Comte, 25000 Besançon, France; 7 Laboratoire MOVE (UR 20296), Faculté des Sciences du Sport, Université de Poitiers, 86000 Poitiers, France; benoit.dugue@univ-poitiers.fr; 8 Department of Surgical Sciences, Physical and Rehabilitation Medicine, University of Torino, 10126 Torino, Italy

**Keywords:** whole-body cryostimulation, obesity, rehabilitation, personalized medicine, gender medicine

## Abstract

**Background**: Whole-body cryostimulation (WBC) involves exposure to extremely low temperatures to reduce inflammation and pain and to enhance recovery. Despite its growing popularity and the importance of the magnitude of WBC-induced skin cooling in triggering the cascade of effects, limited research has focused on skin temperature changes in individuals with severe obesity, where body composition and sex may influence outcomes. **Objective**: To examine differences in the cooling response based on sex and BMI, we conducted an observational study comparing patients with obesity to normal-weight individuals after repeated WBC sessions. The goal was to identify differences in skin temperature drops linked to sex and BMI. **Methods**: A total of 149 adults participated in the study: 119 with obesity (body mass Index, BMI ≥ 30 kg/m^2^) and 30 with normal weight (BMI ≤ 25 kg/m^2^). Participants underwent 10 WBC sessions at −110 °C for 2 min over two weeks. Skin temperatures were measured before and after each session. **Results**: While the overall drop in skin temperature after 10 sessions of WBC was similar between the patients with obesity and normal-weight subjects, significant differences emerged after adjustment for body surface area. Females exhibited a greater decrease in temperature than males in both groups irrespective of BMI. However, among males, normal-weight individuals experienced a significantly greater temperature drop compared to those with obesity. **Conclusions**: The study shows that sex and BMI influence WBC-induced skin temperature changes. The results of this study suggest that WBC protocols should be personalized.

## 1. Introduction

Whole-body cryostimulation (WBC) is a physical/medical treatment based on the exposure of the entire body to very cold temperatures, usually between −110 °C and −140 °C (−166 °F to −220 °F) for a brief period of 2 to 3 min. WBC has become increasingly popular in sports medicine for its potential benefits to athletes [1,2], particularly for its potential to improve recovery [3,4], reduce inflammation [5], manage pain [6], and provide mental benefits [7]. More recently, the use of WBC is also gaining interest in clinical settings. In fact, growing scientific evidence supports the safe clinical use of WBC as an adjuvant treatment in many conditions of rehabilitation interest, including orthopedic, neurological, metabolic, autoimmune, psychiatric, and sleep disorders [8,9,10,11,12]. Its popularity appears to be due to its potentially wide range of benefits. However, there is a lack of research specifically focused on the degree of skin surface cooling induced by WBC, and there is limited consideration of individual characteristics, such as sex and body composition, that may significantly influence individual responses to WBC [13], especially in patients with an excess of body fat.

The available literature on WBC is highly heterogeneous due to variations in the type of cryogenic chambers, exposure protocols, session frequency, and applied temperatures, making comparisons across studies difficult [13,14]. This is an important gap in the knowledge of cold therapies, as skin temperature is the crucial physiological parameter capable of triggering, below a certain threshold [14], a cascade of beneficial physiological and molecular effects. The skin plays a vital role in global homeostasis and body thermoregulation, mediating heat exchange between internal tissues and the external environment. Cutaneous nerves detect environmental temperature, and environmental thermal signals from the skin serve as feedforward signals in the control of body temperature [15,16]. Skin temperature dynamics is regulated by the thermal gradient between the body and the environment, as well as the ability of the tissue to generate heat and attenuate heat loss. This regulation is influenced by factors such as deep tissue adiposity, muscle mass, blood flow, and thermal resistance [13]. The basic premise of cryotherapy is that exposure to cold activates thermosensitive skin receptors, which are the primary cause of cryostimulation efficiency, triggering regulatory mechanisms aimed at maintaining a constant core temperature [17]. Monitoring changes in skin temperature could provide valuable insights into the effectiveness of WBC in stimulating metabolic processes. WBC temperature, duration, and number of sessions represent the main factors determining the “cold dose” administered with cryostimulation, which should also be personalized to the patient’s characteristics that may influence thermal skin variations. Lower temperatures and longer durations of cooling exposure lead to a more intense thermal shock, enhancing physiological responses and potential therapeutic benefits [18]. In this context, evidence suggests specific temperature thresholds, around 12 °C, and a defined reduction range, typically 5–15 °C [19], that are thought to elicit the WBC-associated analgesic response. In fact, decreases in nerve conduction velocity have been observed to be linked to significant reductions in skin temperature, typically in the range of 12.5–13.5 °C [3,18]. The effects of WBC are particularly relevant for obesity, a condition linked to chronic low-grade inflammation. Individuals with obesity may need longer times or colder temperatures of exposure to achieve the same cooling effects as normal-weight individuals, as subcutaneous adipose tissue may act as a thermal insulator just below the interface between the skin and the external environment [20,21,22], limiting the cooling process of the underlying tissues [22]. Studies on humans immersed in cold water have convincingly shown that individuals with obesity cool more slowly and increase their metabolism less significantly than lean individuals to maintain temperature homeostasis [23,24]. Indeed, adipose tissue has lower thermal conductivity than lean tissue, providing an adequately distributed barrier to heat loss [22]. In addition, the accumulation and anatomical localization of adipose tissue also differ between men and women [25], which could partially explain the differences in thermogenic response to cold between sexes before and after the cold stimulus. Several studies showed that sex-related differences, as well as body composition and body surface area-to-mass ratio, can have a significant impact on thermoregulatory responses after WBC [26,27]. According to these studies, conducted in the normal/overweight population, it seems that females exhibit lower skin temperatures immediately after WBC compared to males, with notable differences persisting up to 10 min post-exposure [26]. Despite females storing more of their adipose tissue in the subcutaneous and femoral region, while males store the majority of their white adipose tissue in the abdominal or visceral regions, females experience a faster and more pronounced drop in body temperature after cold exposure. This is likely because males, having more lean muscle mass, generate more heat through shivering, which enhances their response to cold [22].

Understanding the impact of sex and body composition on skin cooling after WBC is crucial for developing personalized treatments, particularly for individuals with moderate-to-severe obesity. These factors may require higher “doses of cold” to penetrate adipose tissue and achieve the therapeutic skin cooling threshold of 12 °C to 13.6 °C [13,18]. This underscores the importance of developing customized, sex-specific WBC protocols considering factors such as optimal session duration, frequency, and temperature settings to maximize benefits and minimize risks.

To examine differences in cooling response based on sex and BMI, we conducted an observational study comparing individuals with obesity to normal-weight individuals after repeated WBC sessions. The goal was to identify skin temperature drop differences and develop WBC protocols that are both effective and safe for varied patient profiles.

## 2. Materials and Methods

### 2.1. Participants

Between June 2021 and March 2023, adult inpatients admitted to the San Giuseppe Hospital, Istituto Auxologico Italiano in Piancavallo (Verbania, Italy), were enrolled in the study. Patients were provided with complete information about the scope and methodology of the study, which was conducted in conformity with the Declaration of Helsinki of the World Medical Association and approved by the Ethics Committee of the Istituto Auxologico Italiano (reference: 2021_05_18_14). Written and verbal informed consent was obtained from all patients.

The sample analyzed in this study represents a subset of a larger cohort originally collected to investigate broader trends in the impact of WBC in patients with metabolic or neurological diseases or fibromyalgia, or healthy normal-weight/overweight patients (study registration: NCT05443100). The subgroup selected for this specific analysis was chosen based on the presence of a diagnosis of obesity and was compared with a sample of normal-weight volunteer hospital staff. Patients with severe obesity and normal-weight volunteers were recruited as two distinct groups for comparison. The final sample size was determined based on the availability of eligible participants during the recruitment period.

### 2.2. Study Design

This study is an observational case-control trial designed to assess the effects of whole-body cryostimulation (WBC) on skin temperature drop in individuals with severe obesity compared to normal-weight subjects. The study is based on the hypothesis that sex and BMI may influence skin temperature responses to WBC.

Adult inpatients (age > 18 years) hospitalized for obesity rehabilitation were considered. These patients underwent a 4-week comprehensive, multidisciplinary rehabilitation program. The comprehensive rehabilitation program included individualized nutrition, psychological support, and supervised physical activities. The patients followed a balanced, hypocaloric Mediterranean diet with specified macronutrient distribution. They underwent two daily 60-min physiotherapy sessions consisting of progressive aerobic training, postural exercises, and strength training, which were monitored by a physiotherapist and adapted to individual fitness and clinical status. During the rehabilitation program, eligible patients were selected for a 10-session cycle of WBC. According to the international consensus on WBC contraindications [28], severe psychiatric conditions, active neoplasia or a recent history of malignancy, acute respiratory disease, acute cardiovascular disease, unstable hypertension, systemic lupus erythematosus, cold agglutinin disease, arthritis, psoriasis, asthma, claustrophobia, pregnancy and lactation, recent modification of usual drug treatment, previous treatment with WBC in the last year, weight loss > 10% in the last 3 months, and body temperature greater than 37.5 °C were considered exclusion criteria for WBC sessions.

### 2.3. Anthropometric Assessments

Body weight (Kg) and body height (meters) were measured with precision to the nearest 0.1 kg and 0.5 cm, respectively. A mechanical column scale (Scale-Tronix, Wheaton, IL) and a stadiometer (Scale-Tronix, Wheaton, IL, USA) were used for these measurements. Body mass index (BMI) was then calculated by dividing body weight by the square of the height (kg/m^2^). The group of patients with obesity (OB) had a BMI ≥ 30 kg/m^2^. A BMI between 30–34.9 kg/m^2^ refers to class I OB, between 35–39.9 kg/m^2^ to class II OB, and ≥40 kg/m^2^ in class III. The waist circumference (cm) was measured with non-elastic tape at the level of the umbilicus. Body composition analysis was performed using a single-frequency bioimpedance analyzer (BIA 101, Akern^®^, Pisa, Italy), as previously described [29]. The body surface area (BSA) was calculated using the DuBois and DuBois formula [30].

### 2.4. Description of the WBC Session and Skin Temperature Assessment

Before the first exposure, all subjects underwent a familiarization session with WBC (T0), which involved a 1-min treatment at −110 °C. They then participated in 10 additional 2-min WBC sessions (T1–T10) over a 2-week period, with one session per day from Monday to Friday at 8:15 a.m., prior to exercise classes and physical therapy. Each patient underwent WBC in a fed state, having consumed a standardized breakfast at least 1 h prior to the WBC. The standardized breakfast consisted of either 175 mL of partially skimmed milk or 125 mL of skimmed yogurt, accompanied by 30 g of low-fat rusks (fat < 12%) or biscuits, providing approximately 350 kcal, corresponding to ~15–20% of the total daily intake.

WBC sessions were performed using a single-person whole-body cryotherapy chamber designed to deliver cryo treatments by liquid nitrogen (Artic, CryoScience, Rome, Italy). All sessions were supervised by specially trained personnel. Skin temperature was measured with a high-precision infrared thermometer with an accuracy of ±1 °C or 1% of the reading, with a distance-to-point ratio of 12:1 (Fluke 62 Max+, Fluke Corporation, Everett, WA, USA) to measure temperatures from −30 °C to +650 °C. Four anatomical reference points were chosen: calf (C), quadriceps (Q), popliteal cavity (PC), and nape of the neck (N). Specifically, skin temperatures were recorded within 1 min immediately before and after each WBC session.

The four reference points are indicative of different cold reactivity due to different underlying muscle thickness (as in the case of C and Q), lower joint soft tissue area (as in the case of PC), and upper body (N). The order of temperature assessment was Q, PC, C, and N on the left half of the body.

The measurement was made by holding the thermometer at a standardized distance of 10–15 cm (corresponding to a skin spot diameter of about 1 cm^2^). To obtain more accurate results, we allowed the thermometer equilibrate to the temperature of the surrounding environment (room temperature), which is consistently maintained at 20–22 °C throughout the year by an air conditioning system, ensuring minimal seasonal fluctuations.

Before entering the cryochamber, subjects were asked to remove glasses, contact lenses, and jewelry, and to dry their bodies of sweat to avoid the risk of cold skin injury. The patient entered the cryochamber minimally dressed, wearing shorts or sweatpants (due to the severe cold sensation in some cases), a light T-shirt or shirtless (or sports bra for women), mid-calf socks, clogs, gloves, headgear, and earmuffs. Importantly, all the previously mentioned reference points were kept uncovered, regardless of clothing choice.

To protect the oral and nasal mucosa from the extreme temperatures, patients were asked to wear a surgical mask. Subjects were instructed to breathe normally in the cryochamber and walk in place or move their fingers to better tolerate the WBC treatment. Visual and verbal contact with patients was maintained throughout the WBC session.

### 2.5. Statistical Analysis

Data were expressed as the mean ± standard deviation (SD) and/or standard error (SE). The deltas were calculated as the difference (Post–Pre WBC sessions) and as the percentage of temperature change (Post–Pre/Pre × 100). The Shapiro-Wilk test was used to assess the normality of the data distribution. Student’s *t*-test for normal data and Wilcoxon’s nonparametric test for non-normal data were used. The repeated-measures ANOVA was used to evaluate changes over time. The model included factors for time points and conditions, with subjects as a random effect to account for repeated measurements. Significant main effects and interactions were reported with *p*-values (significant when <0.05). Post-hoc comparisons were conducted using Tukey’s HSD test, where applicable. All analyses were carried out using the statistical software JMP^®^, Version 16, SAS Institute Inc., Cary, NC, 1989–2023 (USA).

## 3. Results

A total of 149 adult subjects (115 females, 34 males) were considered for this study. Of these, 119 individuals were living with obesity and were hospitalized for a 4-week comprehensive multidisciplinary rehabilitation program. These patients had a body mass index (BMI) ≥ 30 kg/m^2^, with 84% being female and 16% male. The BMI for this group ranged from 30.98 kg/m^2^ to 65.4 kg/m^2^. Fifty percent of patients with OB were in class III (BMI ≥ 40 Kg/m^2^). The normal weight control group (NW) included 63% male and 37% female participants. The mean BMI in this group was 22.9 ± 2.3 kg/m^2^. A summary of demographic and anthropometric data is presented in Table 1.

**Figure 1:**
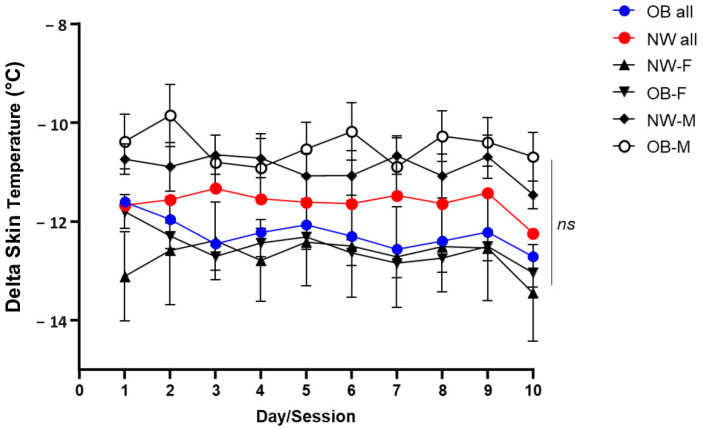
Trend of delta skin temperature across the 10 sessions for subjects with obesity (OB) and normal-weight subjects (NW), analyzed overall (all) and by sex (female, F; male, M). ns *=* no significant differences.

The calculated body surface area (BSA) was 2.12 ± 0.24 m^2^ in the OB group and 1.84 ± 0.16 m^2^ in the NW-CTR group (*p* < 0.0001). The mean BSA was lower in females compared to males in both the OB group (2.06 ± 0.19 m^2^ vs. 2.39 ± 0.18 m^2^, *p* < 0.0001) and the NW group (1.72 ± 0.12 m^2^ vs. 1.91 ± 0.14 m^2^, *p* < 0.0001). Globally, the mean difference in the decrease in skin temperature after 10 sessions was not significantly different between the OB and NW patient groups (respectively: −12.25 ± 2.7 °C vs. −11.61 ± 2.5 °C; *p*-value = 0.06), not significantly different between females with OB and NW (−12.53 ± 2.7 °C vs. −12.71 ± 3.1 °C; *p* = 0.53), nor between males with OB and NW (−10.49 ± 2.1 °C vs. −10.91 ± 1.7 °C; *p* = 0.054) (Figure 1).

**Figure 2:**
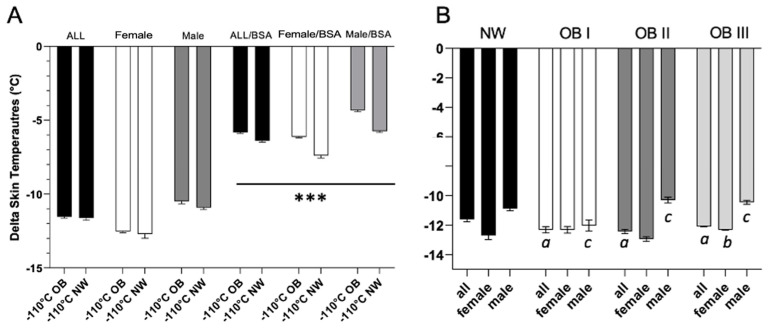
Mean skin temperature drop in patients with obesity (OB) and normal weight (NW) by sex, with and without BSA normalization (**A**), *** = *p*-value < 0.0001. Decrease in skin temperature comparing normal-weight patients (NW) and patients with obesity (OB), classified into three classes of obesity (a = ALL NW vs. ALL OB I; ALL NW vs. OB II; ALL NW vs. OB III; b = Female NW vs. Female OBIII; c = Male NW vs. OBI; Male NW vs. OB II; Male NW vs. OB III) (**B**), a,b,c = *p*-value < 0.0001.

In contrast, significant differences were found when comparing males and females in the OB and NW groups (*p* < 0.0001). The difference between the whole group of OB and NW patients became significant after adjustment for BSA, resulting in −5.84 ± 1.55 °C/m^2^ in OB vs. −6.39 ± 1.58 °C/m^2^ in NW (*p* < 0.0001), and this difference remained significant even after adjustment for BSA by sex (Figure 2A).

Considering obesity classes, we observed a significant temperature drop in females with class II and III obesity compared with males (*p* < 0.05) (Figure 2B).

Table 2 presents the mean temperature decrease at the four anatomical reference points considered: popliteal cavity (PC), nape (N), quadriceps (Q), and calf (C). In the whole group (ALL), the delta skin temperature was significantly different between OB and NW groups at all four reference points, except for the quadriceps (Q). The lowest skin temperature drop observed was in the PC of the OB patient group. In males, the observed temperature drop was statistically different between OB and NW groups (*p* < 0.001), with a greater skin temperature drop at all considered points. On the other hand, we did not observe a significant decrease in overall temperature among females in the OB group compared with the NW group (*p* > 0.05), while the only anatomical reference points showing a significantly greater temperature decrease was the nape of the neck (N) in the NW group. Considering sex, female subjects in both the OB and NW groups showed a significantly greater temperature drop at all reference points than male subjects (*p* < 0.0001).

## 4. Discussion

The present study shows that sex and BMI influence WBC-induced skin temperature changes. The results of this study suggest that WBC protocols should be personalized, with males with obesity potentially needing more intense or longer treatments for optimal cooling.

Previous research on WBC has primarily focused on parameters such as skin temperature magnitude, cardiovascular responses, and subjective thermal comfort, exploring the effects of different WBC durations and temperatures on various populations, including athletes and healthy individuals [31,32]. Moreover, several studies have attempted to establish therapeutic lowering of skin temperature, identifying effective cooling levels below 13.6 °C/12 °C following exposure to WBC, a temperature threshold suspected of triggering the beneficial effects of cold therapy [18,33,34,35,36,37]. However, to date, no study has directly investigated the dynamics of WBC-induced skin cooling in subjects with different degrees of obesity. It has been suggested that tissue adiposity, related to the percentage of fat mass, is a crucial factor influencing the thermal resistance of the body [27,38]. This needs to be taken into consideration when formulating appropriate and sex-specific protocols. For example, women have a greater volume of adipose tissue than men and therefore have greater thermal insulation and thermal resistance, which corresponds to less heat dissipation. In fact, the study by Polidori et al. [39] shows that in healthy, normal-weight subjects, to achieve the same skin temperature effects and thus cool tissues as efficiently, the duration of cryostimulation protocols would have to be shorter for women than for men [13,39]. Consequently, while the study by Polidori et al. provides guidance on the need for shorter durations of WBC for women, there is a significant need for research on how these protocols should be adapted for individuals with obesity.

It is not yet known whether skin temperature in populations with obesity decreases comparably to that in normal-weight individuals. In addition, a precise protocol of efficacy has not yet been identified.

This study is the first to explore the impact of WBC on skin cooling dynamics in a cohort of patients with obesity, potentially providing valuable insights into therapeutic cooling levels for individuals with obesity.

Our results indicate that 10 sessions of WBC at −110 °C affect OB patients differently from NW subjects, but only in males. Skin temperature lowering was significantly different between OB and NW after adjustment for body surface area (BSA), with females showing greater temperature lowering than males in both NW and OB. While no noteworthy differences were observed between OB and NW females, lean NW male subjects exhibited a significant drop in skin temperature at all four anatomical landmarks considered as were otherwise more responsive even after the first WBC. We also observed significant fluctuations in temperature mainly in the lower limbs and did not notice any habituation effect during repeated WBC sessions over the time considered. In summary, 10 sessions of WBC at −110 °C are more effective in females than in males, regardless of BMI status, suggesting that the duration of the cold stimulus should be longer for males than for females, as the latter have faster cooling kinetics than males, confirming evidence from previous WBC studies (conducted in NW subjects) regarding the influence of sex on the level of skin cooling, which showed greater skin drop in females than in males [13].

Females are more sensitive to environmental thermal conditions and adapt more rapidly to exposure to extreme cold air, so they cool more abruptly during cold stress than males [39]. The observed differences between males and females can be explained by anthropometric and thermoregulatory differences: Females generally have lower body mass and body surface area, more fat, greater peripheral vasoconstriction, and reduced sensitivity to cold response [27,38,40]. In contrast, males, characterized by greater lean body mass and thus greater metabolic heat production, might show comparatively higher skin temperatures following WBC. A recent study demonstrated that overweight men reached the analgesic threshold of 13.6 °C in a shorter time when compared to normal-weight men, hence suggesting that WBC protocol should be optimized based on BMI [41].

Therefore, to induce an equivalent skin thermal response, the duration of a single session for males must be longer than that for females. This raises questions about the current clinical practices of centers using WBC, which often adopt identical cryostimulation protocols for males and females.

## 5. Limitations

The study used a very accurate infrared thermometer to measure skin temperature; however, the use of a thermal imaging camera, considered the gold standard in this type of assessment, would have provided more accurate data to map temperature changes in different regions of the body, capturing more subtle changes that could have led to more accurate and reliable conclusions. In addition, because of the imbalance between males and females in the studied cohort, and the relatively low sample size of the normal-weight group, the results may not fully represent the general population and should be replicated. Additional confounding factors that might influence skin temperature responses, such as time of year, hormonal profile, metabolic rate or stress/inflammation, were not considered and deserve further investigation.

## 6. Conclusions

Cutaneous thermal response can be considered a key parameter to assess the impact of sex in cryostimulation and facilitate adjustment of the duration of the protocol required to achieve, for example, −12 °C cooling in a specific region of the body.

Our results support the evidence that males require a different “dose” of cold than females to achieve the same level of skin cooling and provide further evidence that this is maintained even when considering subjects with obesity.

The results of this study thus highlight the need to consider differences in BMI, as well as sex, to achieve the best possible results and ensure a personalized approach to WBC. Currently, WBC protocols do not adequately account for these differences, which may lead to less effective outcomes for patients undergoing this treatment. Personalization of WBC protocols could improve the effectiveness of treatment by ensuring that each subject receives an optimal dose of cold suited to his or her body and physiological characteristics. This personalized approach could lead to greater therapeutic efficacy and an overall improvement in patient experience.

## Figures and Tables

**Figure 3:**
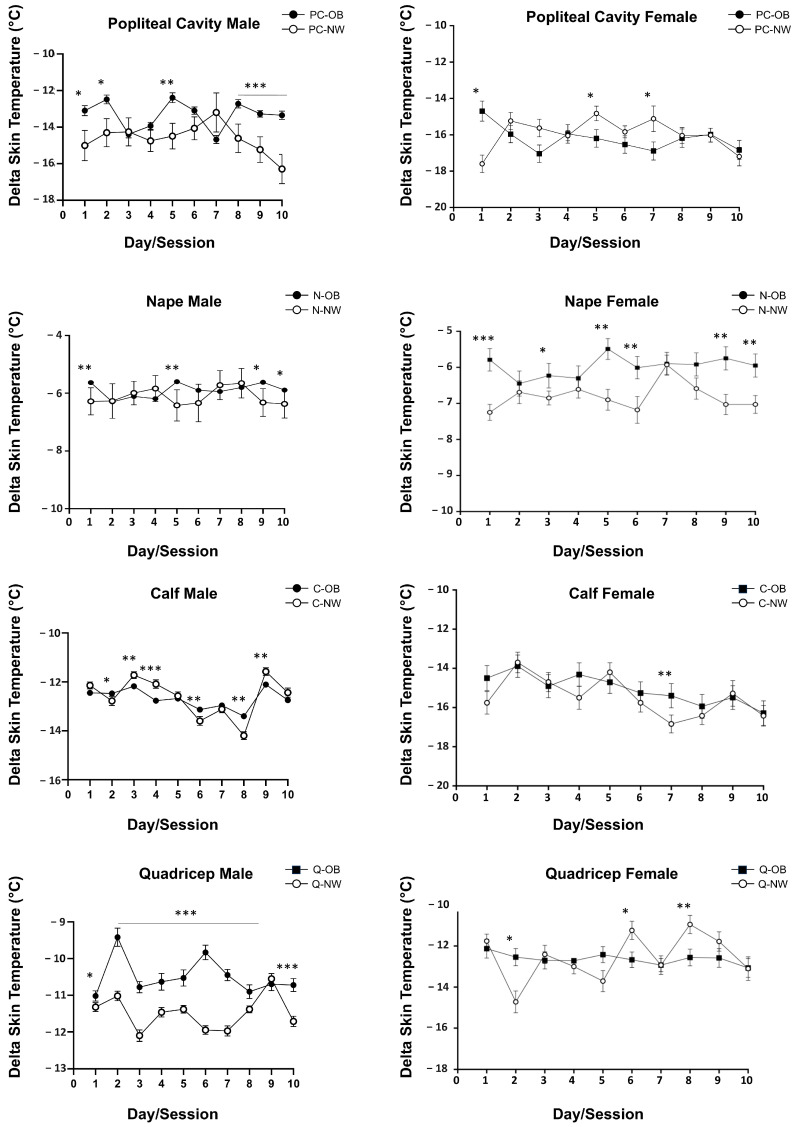
Daily time course of temperature drops during the 10 WBC sessions at the four reference points considered for male and female subjects with obesity (OB) and normal weight (NW). * *p*-value < 0.05; ** *p*-value < 0.01; *** *p*-value < 0.0001.

**Table 1:** Demographic and anthropometric data of the studied cohort.

	OB	NW
N	119	30
Age (years)	56.2 ± 11.7	58.5 ± 13.3
Males (%)	16%	63%
Females (%)	84%	37%
BMI (Kg/m^2^)	41.1 ± 5.6	22.9 ± 2.3
Waist Circumference (cm)	119 ± 11.9	96 ± 1.1
Fat Mass (%)	51.2 ± 5.7	29.2 ± 3.8
Muscle Mass (%)	26.9 ± 5.9	42.2 ± 1.6

OB: patients with obesity, NW: patients with normal weight; BMI: body mass index. Data are expressed as mean ± standard deviation.

**Table 2:** Mean temperature drop (ΔT) at four reference points—popliteal cavity (PC), nape (N), quadriceps (Q), and calf (C)—for patients with obesity (OB) and normal weight (NW) in the whole cohort and by sex. Data are shown as mean and standard deviation (SD). Significant *p*-values are indicated in bold.

	PC			N			Q			C		
	Mean	SD	*p*-Value	Mean	SD	*p*-Value	Mean	SD	*p*-Value	Mean	SD	*p*-Value
ALL OB	−15.82	4.79	**0.0002**	−5.90	3.08	**0.03**	−12.35	4.01	ns	−14.75	5.48	**0.005**
ALL NW	−14.65	4.13		−6.12	2.69		−11.9	3.84		−13.73	4.78	
Female OB	−15.57	4.89	ns	−5.76	3.01	**0.001**	−12.16	4.21	ns	−14.63	5.58	ns
Female NW	−15.95	4.96		−6.8	2.94		−12.56	4.09		−15.46	15.07	
Male OB	−11.34	4.07	**0.001**	−4.43	2.71	**0.001**	−9.34	2,98	**0.001**	−11.79	3.08	**0.03**
Male NW	−13.8	3.24		−5.68	2.42		−11.48	2.09		−12.62	3.76	

The detailed time course of temperature drops during the 10-day WBC session for the four considered reference points is shown in Figure 3. Despite the high variability, in the male group, we observed a significant difference in delta skin temperatures at all four reference points considered and, in particular, it was lower in NW individuals even after the first WBC session, especially in popliteal cavity (PC) and nape (N). In the quadriceps (Q), NW males were more responsive to the stimulus than OB males starting from day 2 of WBC exposure. In the NW female group, the nape (N) was the most responsive site when compared to the values observed in OB female patients. ns *=* no significant differences.

## Data Availability

The data supporting the findings of this study are available from the corresponding author upon reasonable request.
